# No causal association between tea consumption and 7 cardiovascular disorders: A two-sample Mendelian randomization study

**DOI:** 10.3389/fgene.2022.989772

**Published:** 2022-11-30

**Authors:** Dongsheng Cai, Jun Chen, Yuteng Wu, Chenyang Jiang

**Affiliations:** ^1^ Department of Cardiology, Sir Run Run Shaw Hospital, School of Medicine, Zhejiang University, Hangzhou, China; ^2^ The First Clinical College, Zhejiang Chinese Medical University, Hangzhou, China

**Keywords:** tea consumption, causal association, Mendelian randomization (MR), SNPs (single nucleotide polymorphisms), atrial fibrillation

## Abstract

**Background:** Previous studies have reported inconsistent results on the causal association between habitual tea consumption and the risk of cardiovascular disease (CVD). This study is aim to determine the association between habitual tea intake and CVD using two-sample Mendelian randomization (MR) analysis.

**Methods:** The genetically predicted causation between tea consumption and 7 common cardiovascular diseases (atrial fibrillation, hypertension, acute myocardial infarction, coronary atherosclerosis, peripheral vascular disease, angina, and heart failure) was evaluated using MR analysis model. We performed a total of 9 MR analysis methods to analyze the final results. The IVW methods was used as the primary outcome. The other MR analysis method (simple mode, weighted mode, simple median, weighted median, penalized weighted median, MR Egger, and MR-Egger (bootstrap)) were performed as the complement to IVW. Also, the robustness of the MR analysis results was assessed using a leave-one-out analysis.

**Results:** The IVW analysis methods indicated that there is no causal association between tea consumption and risk of CVD (AF: OR, 0.997, 95% CI, 0.992–1.0001, *p* = 0.142; hypertension: OR, 0.976, 95% CI, 0.937–1.017, *p* = 0.242; AMI: OR, 0.996, 95% CI, 0.991–1.000, *p* = 0.077; CA: OR, 1.001, 95% CI, 0.993–1.009, *p* = 0.854; PVD: OR, 1.002, 95% CI, 1.000–1.005, *p* = 0.096; angina: OR, 0.999, 95% CI, 0.993–1.006, *p* = 0.818; HF: OR, 0.999, 95% CI, 0.996–1.002, *p* = 0.338). The other MR analysis method and further leave-one-out sensitivity analysis suggested the results were robust.

**Conclusion:** This MR study indicated that there was no genetically predicted causal association between habitual tea intake and risk of CVD.

## Introduction

Cardiovascular disease (CVD) is the leading cause of death worldwide, accounting for one-third of global mortality (Roth et al., 2020). Considering the substantial global burden of CVD, there is a great interest in identifying potential preventative approaches. It has been shown in previous studies that dietary factors may play an important role in the development of CVD (Kim et al., 2019; Shin et al., 2022).

Tea culture is popular all over the world and tea is currently the best-selling beverage worldwide. The causal relationship between habitual tea intake and the risk of CVD is still unclear. On one hand, tea contains phenolic compounds, with anti-oxidant and anti-inflammatory activities, which may contribute to cardiac protection (Manolis et al., 2022). On the other hand, the presence of the substantial amount of caffeine in tea may cause cardiac arrhythmia (Voskoboinik et al., 2019). So far, several studies reported that habitual tea intake may reduce the risk of CVD (Wang et al., 2020; Niu et al., 2021; Chen et al., 2022). However, other observational studies suggested that tea consumption may be associated with a higher risk of CVD (Malasevskaia et al., 2020; Feng et al., 2021; Zheng et al., 2022). In addition, there are also several studies indicated that there is no causation between tea consumption and CVD (Wang et al., 2019; Igho-Osagie et al., 2020). In general, the existing results are contradictory and the genetically predicted causal effect of tea consumption on CVD is still uncertain.

The conflicting results come from previous observational studies are most likely to be attributed to reverse causality and unmeasured confounding. Using genetic variants as instrumental variables (IV), Mendelian randomization (MR) analysis method can eliminate latent confounding factors and reverse causality (Bennett and Holmes, 2017). MR analysis is therefore capable of examining the causal relationship between exposure and outcome in a robust manner. Thus, we conducted a two-sample MR analysis to explore the causation between tea consumption and the risk of CVD in this study.

## Methods

### Study design and data sources

**FIGURE 1:**
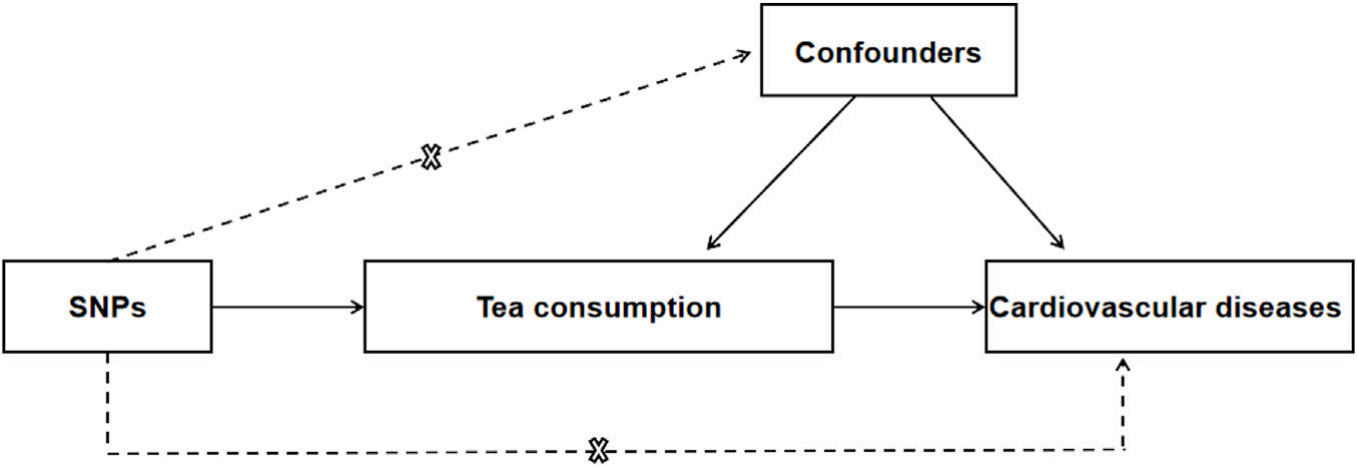
Mendelian randomization model of tea consumption and risk of CVD. The design is under the assumption that the genetic variants are associated with tea consumption, but not with confounders, and the genetic variants influence CVD only through tea consumption. CVD, cardiovascular disease; SNP, single nucleotide polymorphism.

In contrast to one-sample MR analysis, two-sample MR analysis obtains the effect of IV-exposure association and IV-outcome association from two different sample. In other words, one-sample MR analysis is only possible to estimate the relationship between genetic variants and exposure outcomes in one sample. As opposed to one-sample MR, two-sample MR can analyze the association in much larger sample size and therefore obtain statistically higher precision (Lawlor, 2016). Thus, a two-sample MR analysis was conducted in this study to evaluate the causal effect of tea consumption on 7 cardiovascular diseases, including atrial fibrillation (AF), hypertension, acute myocardial infarction (AMI), coronary atherosclerosis (CA), peripheral vascular disease (PVD), angina, and heart failure (HF) (Figure 1). The single nucleotide polymorphisms (SNPs) of both habitual tea consumption and CVD were derived from recently published genome-wide association studies (GWAS). Detailed information about data sources is shown in Table 1. The GWAS meta-analyses data had obtained relevant ethical approval from their ethics committee. In this study, only the summarized data extracted from the GWAS database was used. Therefore, no additional ethics approval is needed for this study.

**TABLE 1:** Descriptions for data sources and assessment of the instrumental variables strength.

**Traits**	**Data sources (ID)**	**Sample size**	**Cases**	**Controls**	**Ancestry**	**F-statistic**	**Access link**
Exposure							
Tea intake	United Kingdom Biobank (ukb-b-6066)	447,485			European		https://gwas.mrcieu.ac.uk/datasets/ukb-b-6066
Outcome							
AF	United Kingdom Biobank (ukb-a-536)	337,199	3,818	333,381	European	16.879	https://gwas.mrcieu.ac.uk/datasets/ukb-a-536
Hypertension	United Kingdom Biobank (ukb-a-61)	337,159	87,690	249,469	European	15.984	https://gwas.mrcieu.ac.uk/datasets/ukb-a-61
AMI	United Kingdom Biobank (ukb-a-533)	337,199	3,927	333,272	European	24.203	https://gwas.mrcieu.ac.uk/datasets/ukb-a-533
CA	United Kingdom Biobank (ukb-d-I9)	361,194	14,334	346,860	European	5.652	https://gwas.mrcieu.ac.uk/datasets/ukb-d-I9
PVD	United Kingdom Biobank (ukb-b-4929)	463,010	1,456	461,554	European	34.851	https://gwas.mrcieu.ac.uk/datasets/ukb-b-4929
Angina	United Kingdom Biobank (ukb-b-8650)	462,933	14,770	448,163	European	10.161	https://gwas.mrcieu.ac.uk/datasets/ukb-b-8650
HF	United Kingdom Biobank (ukb-d-I50)	361,194	1,088	360,106	European	13.368	https://gwas.mrcieu.ac.uk/datasets/ukb-d-I50

AF: atrial fibrillation; AMI: acute myocardial infarction; CA: coronary atherosclerosis; PVD: peripheral vascular disease; HF: Heart failure.

### Single nucleotide polymorphisms selection

In this Study, genetic variants were selected as IV. IV should meet the following MR assumptions: 1) There was a strong association between exposure and IV. 2) There was no association between IV and any confounders. 3) IV directly impacted the outcomes *via* exposure instead of other pathways. Tea consumption data was derived from the recently published GWAS analyzing 447,485 individuals of European ancestry of tea consumption based on United Kingdom Biobank. Autosomal biallelic SNPs with *p*-value <5 × 10^–8^ and minor frequency >1% were selected as preliminary candidate SNPs (2,672 unique SNPs). Furthermore, the independence of the chosen genetic variants was confirmed through clumping these 2,672 SNPs with linkage disequilibrium (*r* ^2^ < 0.001 at a 10,000 kb window). Then the association between SNPs and established confounders (such as smoking, lipid traits, obesity, and diabetes) was examined by “PhenoScanner” (http://www.phenoscanner.medschl.cam.ac.uk/). Ultimately, we found 49 independent SNPs related to tea consumption. The SNPs data for CVD were derived from the recently published largest GWAS (https://gwas.mrcieu.ac.uk/) (Nielsen et al., 2018). Detailed information of all SNPs used in this study are presented in Supplementary Table S1–7.

### Statistical analysis

This study was performed using a total of 9 MR analysis methods: inverse-variance weighted approach (IVW) (fixed-effect and random-effect model) (Bowden et al., 2016), MR-Egger regression approach (General model and bootstrap model) (Bowden et al., 2015), weighted median estimator analyses (simple median, weighted median, and Penalised weighted median) (Bowden et al., 2016), and mode based-estimator analyses (weighted mode based-estimator analyses and simple mode based-estimator analyses) (Hartwig et al., 2017). IVW was the primary method to analyze the results because it can provide robust causal estimate even when heterogeneity exists. Due to the requirement that all instrumental variables should meet the MR assumptions in the IVW method, the other methods were used as the complement to IVW. The MR-Egger regression could detect and adjust the pleiotropy, and obtain a causal effect assessment and determine whether directional horizontal pleiotropy is responsible for the results. The weighted median estimator could provide consistent causal assess when more than half of the instrumental variables are valid. The mode based-estimator method can reduce the bias and therefore are less biased than other methods but were less precise. Furthermore, we used IVW methods with MR Egger intercept (Burgess and Thompson, 2017) and Cochran’s Q statistics to assess the pleiotropy and heterogeneity of individual SNPs. As long as the intercept did not significantly differ from 0 (*p* > 0.05), pleiotropic effects were considered absent. The value of Cochrane’s Q was used to evaluate the heterogeneity. When the *p*-value was <0.05, the IVW method with multiplicative random-effects model was applied as the primary outcome; otherwise, the IVW method with fixed-effects model was used as the primary outcome. In this study, the F-statistic was applied to assess the strength of the IV–exposure association. F-statistics was calculated as (N-k-1)/k*R ^2^/(1-R^2^). *R* ^2^: the cumulative explained variance of selected SNPs on exposure; k: the number of selected SNPs; N: the sample size. In the case of F > 10, the association between IV and exposure was considered strong enough. Moreover, a leave-one-out analysis was conducted to assess the robustness of MR analysis results through any outlier SNP. Referring to the previous study (Zhang et al., 2021), the causal relationship was deemed significant if three conditions were met: 1) The *p*-value of IVW <0.05.2) There is no difference in the direction of estimates by the IVW, MR-Egger, and weighted median methods. 3) The *p*-value of MR-Egger intercept test was more than 0.05. We used the ‘TwoSampleMR’ R package for all MR analyses involved in this study. All Statistical analyses were performed by R version 3.4.2 (R Foundation for Statistical Computing, Vienna, Austria), and a two-tailed *p*-value < 0.05 was statistically significant.

## Results

**FIGURE 2:**
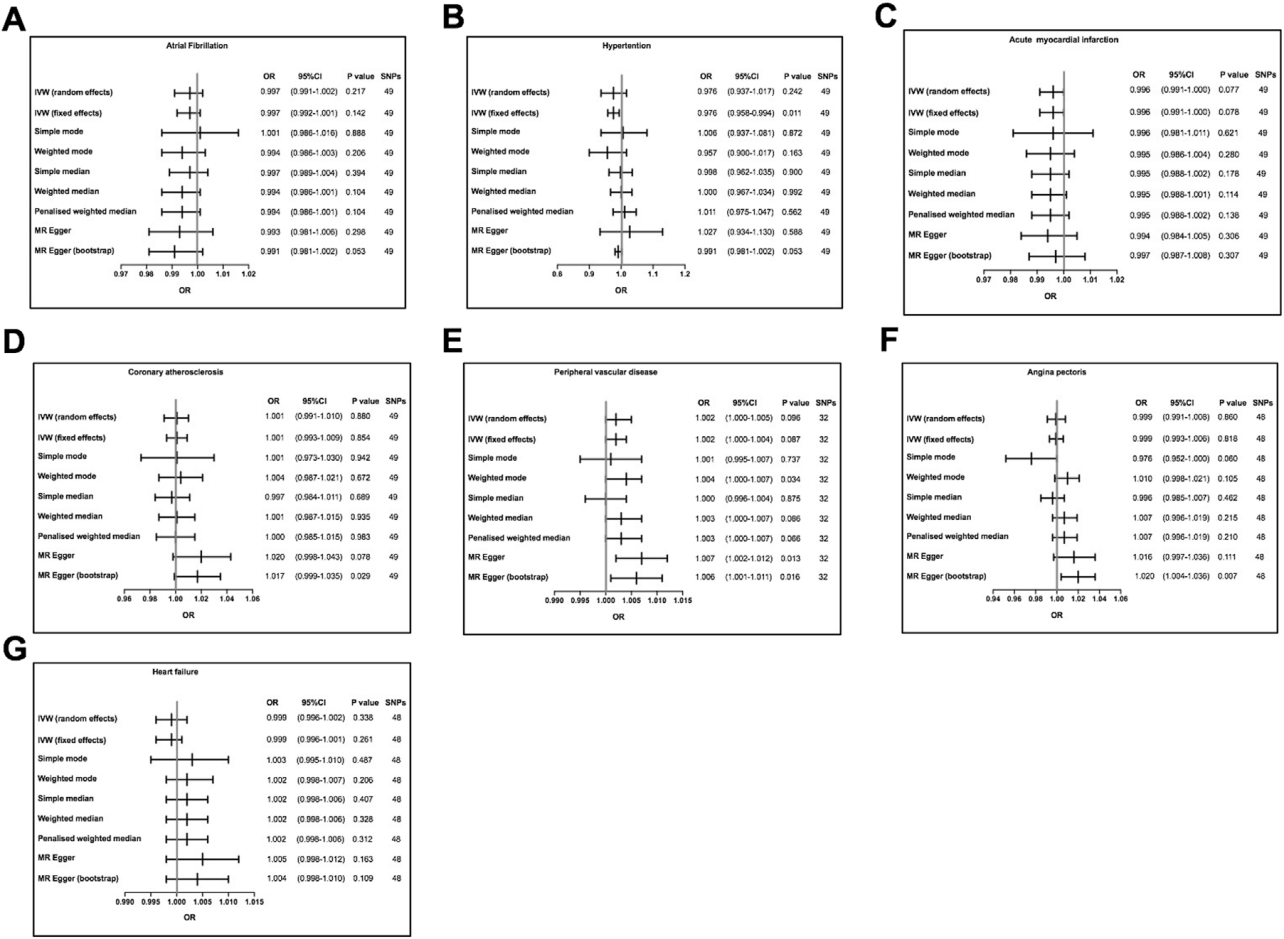
Mendelian randomization assessment of the association between tea consumption and the risk of CVD. **(A)** atrial fibrillation; **(B)** hypertension; **(C)** acute myocardial infarction; **(D)** coronary atherosclerosis; **(E)** peripheral vascular disease; **(F)** angina; **(G)** heart failure. The slope of the straight line indicates the magnitude of the causal association. SNPs, single nucleotide polymorphisms; OR, odds ratio; CI, confidence interval.

**FIGURE 3:**
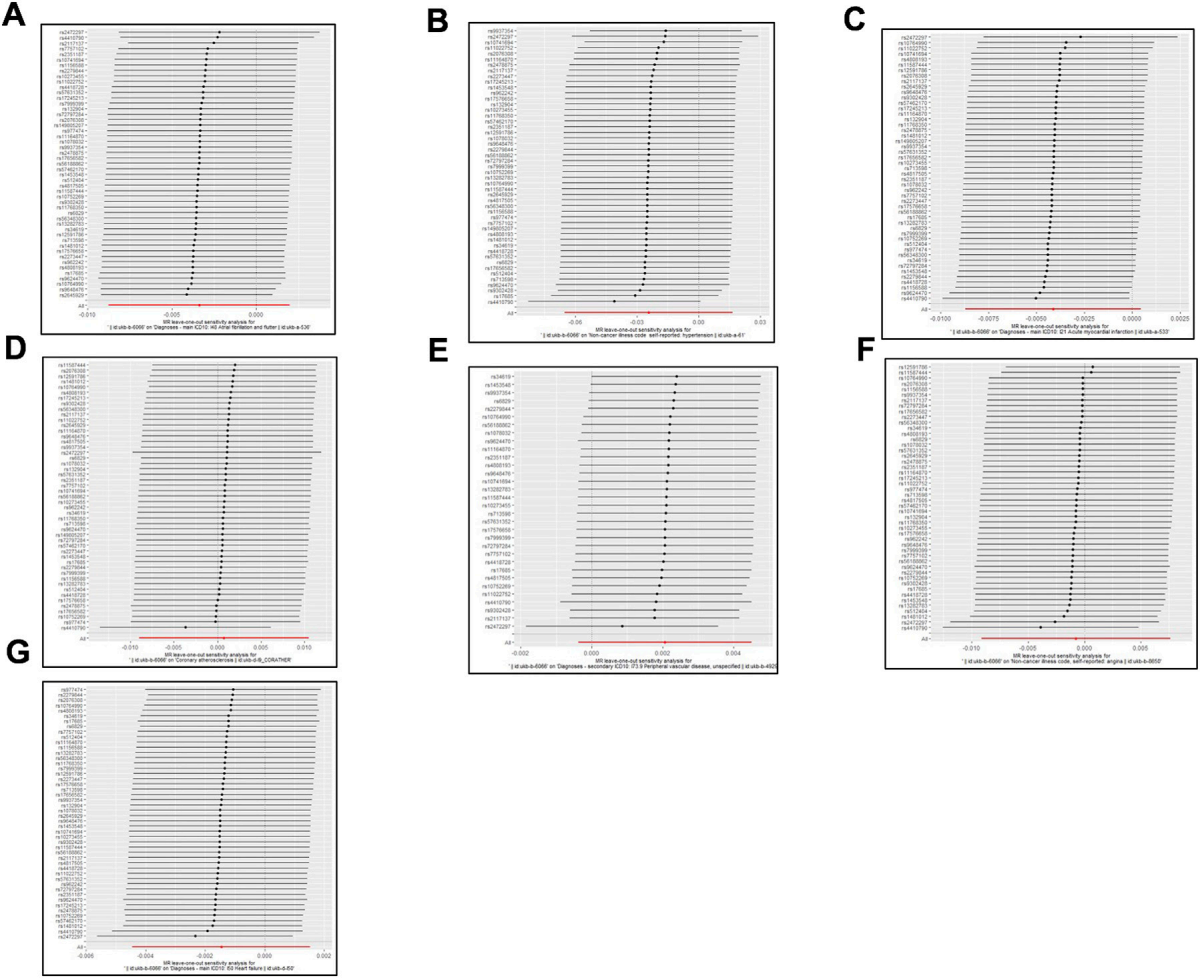
MR leave-one-out sensitivity analysis for tea consumption on CVD. **(A)** atrial fibrillation; **(B)** hypertension; **(C)** acute myocardial infarction; **(D)** coronary atherosclerosis; **(E)** peripheral vascular disease; **(F)** angina; **(G)** heart failure. Circles indicate MR estimates for tea consumption on CVD using inverse-variance weighted fixed-effect method if each SNP was omitted in turn. The bars indicate the CI.

**FIGURE 4:**
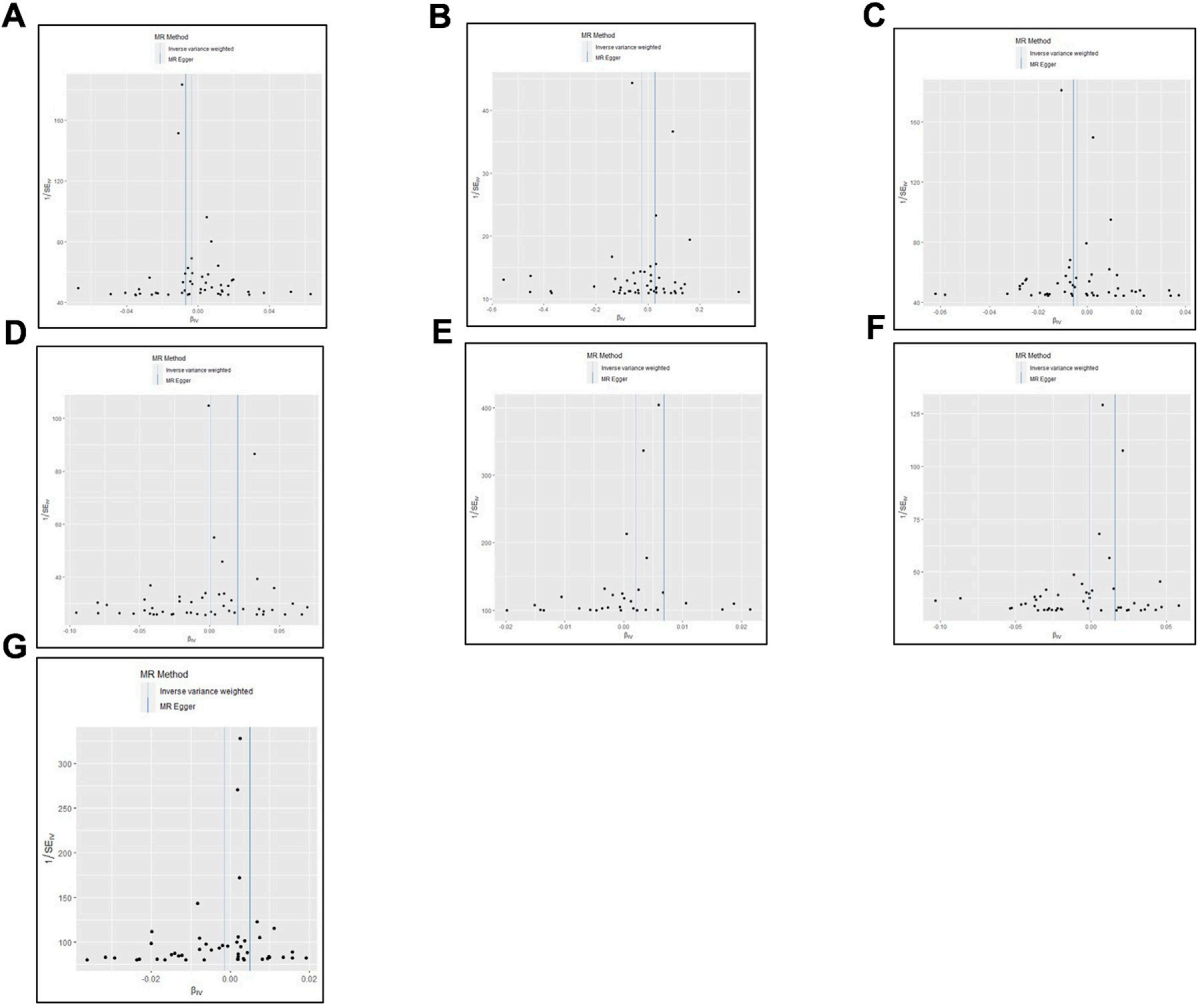
The estimation of heterogeneity using Funnel plot. **(A)** atrial fibrillation; **(B)** hypertension; **(C)** acute myocardial infarction; **(D)** coronary atherosclerosis; **(E)** peripheral vascular disease; **(F)** angina; **(G)** heart failure.

Detailed descriptions of all genetic datasets used in this study are presented in Table 1. As shown in Table 1, the majority F statistics for genetic instruments are greater than 10, therefore, there is less likelihood of weak instrumental bias in this study. As presented in Figure 2 and Supplementary Figure S1, a total of 9 MR methods were performed to analyze the final results. The IVW methods as the primary outcome indicated that there is no causal association between tea consumption and risk of CVD (AF: OR, 0.997, 95% CI, 0.992–1.0001, *p* = 0.142; hypertension: OR, 0.976, 95% CI, 0.937–1.017, *p* = 0.242; AMI: OR, 0.996, 95% CI, 0.991–1.000, *p* = 0.077; CA: OR, 1.001, 95% CI, 0.993–1.009, *p* = 0.854; PVD: OR, 1.002, 95% CI, 1.000–1.005, *p* = 0.096; angina: OR, 0.999, 95% CI, 0.993–1.006, *p* = 0.818; HF: OR, 0.999, 95% CI, 0.996–1.002, *p* = 0.338), as shown in Figure 2. The other MR analysis method (simple mode, weighted mode, simple median, weighted median, penalized weighted median, MR Egger) were performed as the complement to IVW, confirming the robustness of the IVW analysis results (Figure 2 and Supplementary Figure S1). As summarized in Supplementary Table S8, the pleiotropy and heterogeneity of individual SNPs were examined by IVW methods, MR Egger intercept, and Cochran’s Q statistics. The results of the leave-one-out sensitivity analysis indicated that the association between tea consumption and CVD risk was not substantially driven by any individual SNP (Figure 3). The funnel plot result showed that there was no evidence of obvious heterogeneity across the estimates (Figure 4).

## Discussion

In this study, we used up to 9 MR analysis methods to estimate the association between tea consumption and risk of CVD in a large sample size. Our data showed that there is no relationship between tea consumption and the risk of 7 common cardiovascular diseases, including atrial fibrillation, hypertension, acute myocardial infarction, coronary atherosclerosis, peripheral vascular disease, angina, and heart failure.

Due to the popularity of habitual tea intake and the high morbidity of CVD around the world, it is of great significance to clarify the role of tea consumption on CVD. However, previous studies exhibited inconsistent results. There are several possible reasons. First, reverse causality and residual confounding cannot be excluded from previous observational studies. For example, the effect of other anti-oxidative ingredients in food, which have been shown to be associated with the risk of CVD, has not been adjusted in these studies (Rodrigo et al., 2013; Yuan and Larsson, 2019). In addition, compared with people who work during the day, individuals who work night shifts may prefer habitual tea intake because tea drinking may help them improve their attention and work better during the night. Nevertheless, this reverse day/night lifestyle is also a potential confounding of cardiac arrhythmia. Second, the data from previous studies were obtained based on self-reported tea intake. There may exist a misclassification of habitual tea intake. Therefore, the measurement of long-term tea consumption in observational studies may be inaccurate (Liu et al., 2016). Last, in real-world settings, habitual tea-drinking populations are not randomly distributed. It has been influenced to a certain extent by the popularity of tea culture in different areas. It also has a close relationship with age and gender. All of these factors may influence the results of previous observational studies.

The greatest advantage of this study is the two-sample MR study design. MR is a genetic epidemiology design, which can conquer unmeasured confounding and make more robust causal inferences. Thus MR is a particularly powerful approach to access whether an exposure factor has a causation with the development of one disease. In our study, the association between tea consumption and CVD was fully estimated using a total of 9 MR methods in a large sample size. Our work contributes new evidence that there is no relationship between habitual tea intake and risk of CVD. To some extent, our work may helpful in further understanding the effects of habitual tea intake on cardiovascular disease and may useful in guiding the dietary management of patients with CVD.

Of note, there are several inevitable limitations. First, the data in this study was obtained from a European database. Thus the finding might not be applicable in other ethnic groups. Second, the effect of tea type and the amount of intake cannot be evaluated because there was no corresponding information in the database. Third, considering the exposure and outcomes were derived from United Kingdom biobank population, there may be a degree of sample overlap. We do not have a good way to evaluate the overlapping sample size in our study. Population overlapping would increase the possibility of false positives. However, our results are negative. Therefore, population overlapping is unlikely to affect our conclusion.

## Conclusion

This MR study suggested that there is no causal relationship between habitual tea intake and risk of CVD.

## Data Availability

The original contributions presented in the study are included in the article/Supplementary Material, further inquiries can be directed to the corresponding authors.
