# Supplementary material for: No causal association between tea consumption and 7 cardiovascular disorders: A two-sample Mendelian randomization study
Source: Front Genet. 2022 Nov 30;13:989772. doi: 10.3389/fgene.2022.989772 (PMC9748479; doi:10.3389/fgene.2022.989772)
Supplement: Supplementary file 1 [file DataSheet1.pdf]

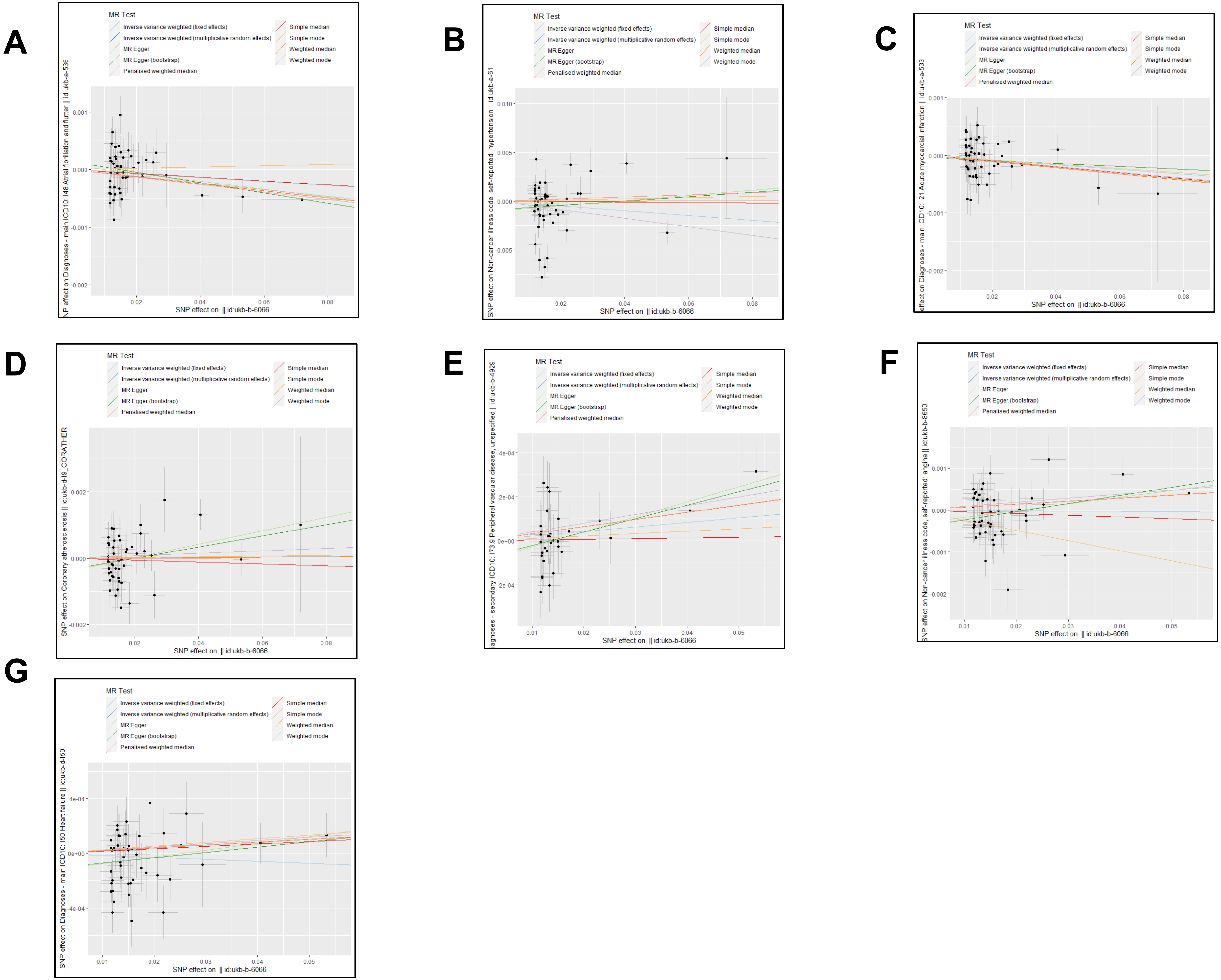

A
MR Test
Inverse variance weighted (fixed effects)
Inverse variance weighted (multiplicative random effects)
MR Egger
MR Egger (bootstrap)
Penalised weighted median
Simple median
Simple mode
Weighted median
Weighted mode
SNP effect on Diagnoses - main ICD10: I48 Atrial fibrillation and flutter || id:ukb-a-536
SNP effect on || id:ukb-b-6066
B
SNP effect on Non-cancer illness code self-reported: hypertension || id:ukb-a-61
SNP effect on || id:ukb-b-6066
C
effect on Diagnoses - main ICD10: I21 Acute myocardial infarction || id:ukb-a-533
SNP effect on || id:ukb-b-6066
D
SNP effect on Coronary atherosclerosis || id:ukb-d-I9_CORATHER
SNP effect on || id:ukb-b-6066
E
agnoses - secondary ICD10: I73.9 Peripheral vascular disease, unspecified || id:ukb-b-4929
SNP effect on || id:ukb-b-6066
F
SNP effect on Non-cancer illness code, self-reported: angina || id:ukb-b-8650
SNP effect on || id:ukb-b-6066
G
SNP effect on Diagnoses - main ICD10: I50 Heart failure || id:ukb-d-I50
SNP effect on || id:ukb-b-6066

Supplement figure 1. Scatter plot to visualize causal effect of tea consumption on CVD.

A: atrial fibrillation; B: hypertension; C: acute myocardial infarction; D: coronary atherosclerosis; E: peripheral vascular disease; F: angina; G: heart failure.
The slope of the straight line indicates the magnitude of the causal association.

Supplement table 1-7. Characteristics of SNPs used for the MR analysis in the summary statistics reported in the GWAS on tea consumption and CVD.

Column A: Number (the order number of SNP)

Column B: SNP (single nucleotide polymorphisms)

Column C: effect_allele.exposure (the main effect allele of single nucleotide polymorphisms in exposure)

Column D: other_allele.exposure (the other effect allele of single nucleotide polymorphisms in exposure)

Column E: effect_allele.outcome (the main effect allele of single nucleotide polymorphisms in outcome disease)

Column F: other_allele.outcome (the other effect allele of single nucleotide polymorphisms in outcome disease)

Column G: beta.exposure (the beta value of single nucleotide polymorphisms in exposure)

Column H: beta.outcome (the beta value of single nucleotide polymorphisms in outcome disease)

Column I: eaf.exposure (the main effect allele frequency of single nucleotide polymorphisms in exposure)

Column J: eaf.outcome (the main effect allele frequency of single nucleotide polymorphisms in outcome disease)

Column K: id.outcome (the ID of outcome disease in GWAS database)

Column L: Chr (the number of chromosome)

Column M: Pos (the locus of chromosome)

Column N: se.outcome (the estimated standard error value of odds ratio for outcome disease)

Column O: samplesize.outcome (the population sample of outcome disease)

Column P: pval.outcome (the p value of odds ratio for outcome disease)

Column Q: outcome (the name of outcome disease)

Column R: se.exposure (the estimated standard error value of odds ratio for exposure)

Column S: pval.exposure (the p value of odds ratio for exposure)

Column T: samplesize.exposure (the population sample of exposure)

Column U: chr.exposure (the number of chromosome in exposure)

Column V: pos.exposure (the locus of chromosome in exposure)

Column W: id.exposure (the ID of exposure in GWAS database)

Supplement Table 8: The results of pleiotropy and heterogeneity test.

variable	Pleiotropy test			Heterogeneity test					
	MR-Egger			MR-Egger			IVW		
	Intercept	SE	P value	Q	Q_df	Q_P value	Q	Q_df	Q_P value
AF	$6.80*10^{-5}$	$1.16*10^{-4}$	0.562	67.34	47	0.027	67.83	48	0.031
Hypertension	-0.001	$8.74*10^{-4}$	0.056	222.10	47	<0.001	228.47	48	<0.001
AMI	$3.13*10^{-5}$	$9.90*10^{-5}$	0.006	47.61	47	0.448	47.71	48	0.484
CA	$-4.00*10^{-4}$	$2.00*10^{-4}$	0.061	66.45	47	0.032	71.66	48	0.015
PVD	$-9.58*10^{-5}$	$4.60*10^{-5}$	0.006	28.43	30	0.548	32.78	31	0.380
Angina	$-3.30*10^{-4}$	$1.8*10^{-4}$	0.067	73.59	46	0.006	79.22	47	0.002
HF	$-1.27*10^{-4}$	$6.27*10^{-5}$	0.049	59.38	46	0.089	64.67	47	0.044

AF: Atrial fibrillation; AMI: Acute myocardial infarction; CA: Coronary atherosclerosis; PVD: Peripheral vascular disease; HF: Heart failure.
